# Abnormal Anatomical and Radiological Changes in a Rare Presentation of Adult Developmental Dysplasia of the Hip

**DOI:** 10.7759/cureus.9938

**Published:** 2020-08-22

**Authors:** Jnana Aditya Challa, Rafid Kasir, Varna Taranikanti

**Affiliations:** 1 Foundational Medical Studies, Oakland University William Beaumont School of Medicine, Rochester, USA; 2 Department of Orthopedic Surgery, Beaumont Health System, Royal Oak, USA

**Keywords:** developmental dysplasia, hip dislocation, radiological changes, treatment dilemma

## Abstract

Adult presentation of bilateral dysplasia and dislocation is an extremely rare presentation. The management of adult hip dysplasia is to preserve the hip and reduce pain through surgical intervention. Hence, early diagnosis provides more options as the treatment dilemma with the late presentation is very complicated with debatable prognosis. The case presented is a 53-year old woman who complained of persistent pain in the hip region. On radiology, dysplasia and dislocation of both the hip joints were observed along with soft tissue abnormalities around the joint. In this case report, we discuss the underlying pathophysiology that might have led to the abnormal radiological and anatomical changes in the hip region and the possible treatment options in a conservatively managed case of developmental dysplasia of the hip (DDH).

## Introduction

Hip dysplasia is a developmental defect of the hip joint where there is anomalous development of the osseous and soft tissue structures around the hip joint [1]. Developmental dysplasia of the hip (DDH) is the most common orthopedic disorder in newborns (1:100) and is more common in first-born females (5F:1M). However, complete dislocation is more commonly seen in adults as compared to children; thus early diagnosis of DDH is extremely important for therapeutic success. There have been very few reports of adult DDH as a consequence of irregular long-term follow-up of childhood DDH [2]. The treatment dilemma with the late presentation is very complicated with a debatable prognosis. Herein we report a 53-year-old woman who presented with persistent pain in the right groin and gluteal region radiating to the distal thigh. Radiology confirmed developmental dysplasia of the hip joint with complete dislocation. In this case report, we discuss the underlying pathophysiology that might have led to the abnormal radiological and anatomical changes in the hip region and the possible treatment options in a conservatively managed case of DDH.

## Case presentation

A 53-year-old woman came to the Beaumont Orthopaedic Institute (BOI) Joints clinic with persistent pain in the right groin and gluteal region radiating to the distal thigh for over a year. The symptoms were not preventing her from doing routine activities. She denied numbness or tingling and has been undergoing physical therapy for this pain for a year, which she reports did not help. Her previous surgical history revealed valgus producing osteotomy with bilateral femoral plates and subsequent removal of the plate on the right side. Physical examination revealed a conscious and mentally alert woman who was able to ambulate without a significant limp. The hip examination findings are shown in Table 1.

**Table 1:** Hip examination findings

	Right	Left
Movements	Hip flexion to 80°; internal rotation 30°; external rotation 30°	Hip flexion to 80°; internal rotation 45°; external rotation 45°
	Knee and ankle normal	
Tone	Soft, supple, and non-tender	
Sensations	Sensations Intact to light touch bilaterally	
Pulses	Peripheral pulses palpable bilaterally	

**Figure 1:**
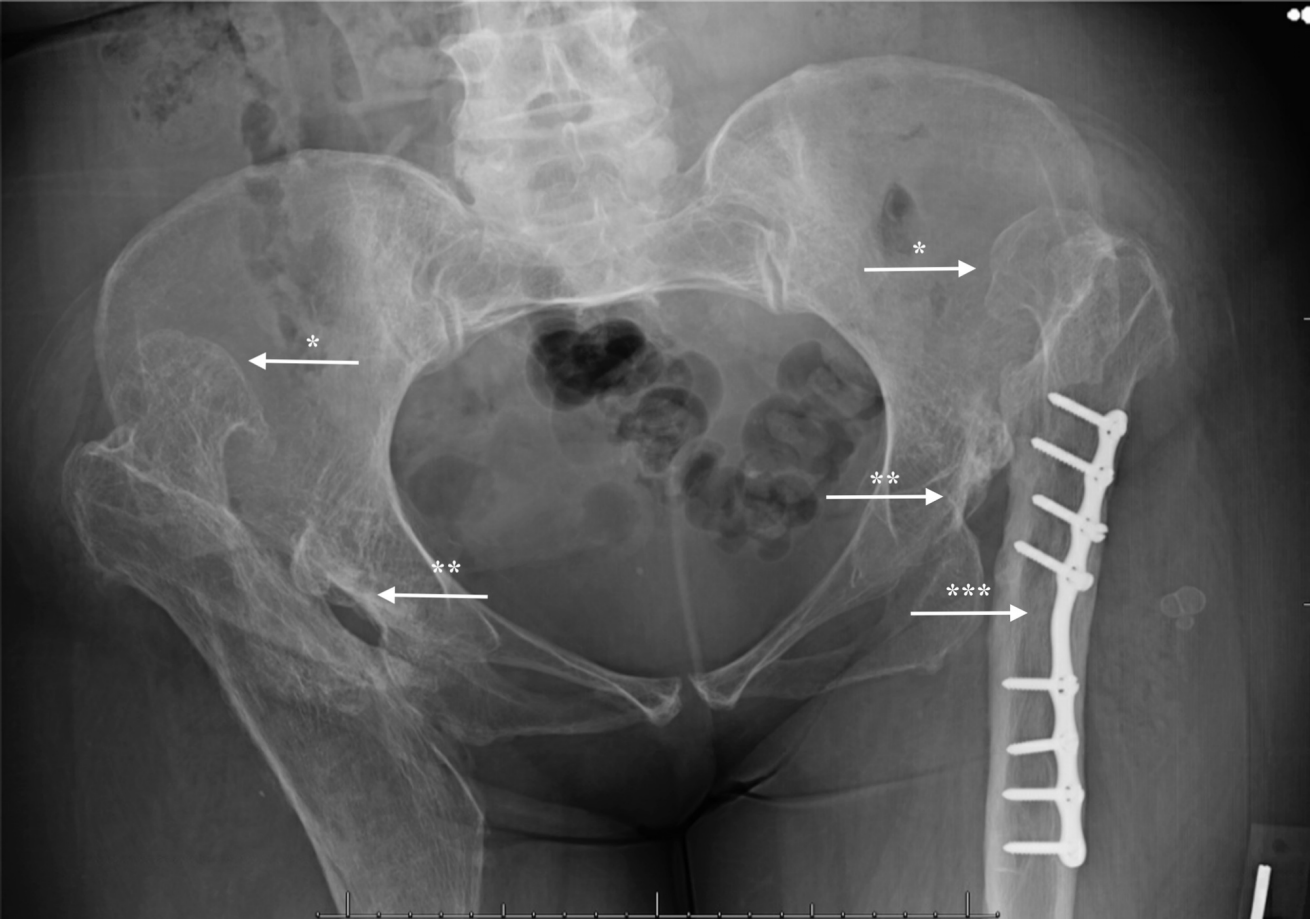
Anteroposterior view showing bilateral femoral dysplasia The femoral head and neck are dysplastic (*) with coxa valga deformity (increased caput collum diaphyseal angle/femoral neck anteversion angle), shallow acetabular socket (**) and femoral plate (***). X-rays: courtesy of Beaumont Royal Oak Hospital.

Reviewed X-rays showed bilateral acetabular hip dysplasia with significant superior dislocation of bilateral femoral heads and advanced degenerative changes. Figure 1 shows anomalous development of the osseous and soft tissue structures around the hips bilaterally. The osseous component involved in the right acetabulum is underdeveloped and defective: it is angular in shape, smaller in size, and decreased in depth.

## Discussion

DDH is a complex disorder mainly caused by the incongruity between the head of the femur and the abnormal socket of the acetabulum. The disease spectrum ranges from mild dysplasia to dislocation of the hip secondary to capsular laxity and mechanical factors (such as movements occurring from hip flexion to extension [3, 4]. 

In this case report, we described a post-surgical patient who has previously been treated with valgus producing osteotomy with bilateral femoral plates for hip deformity. The femoral plate on the right side has since been removed and based on history, physical examination and radiology her condition has been diagnosed as adult dysplasia of the hip.

During embryonic development, limb buds begin to form in the fourth week. In the eighth week of gestation, the hip joint starts developing with the appearance of the acetabulum and femoral head. Soft tissue structures around the hip joint occur during the 12th week and muscles and ligaments start to develop at around 18th weeks. DDH commonly occurs during the last four weeks of fetal growth and perinatally because of mechanical forces acting on the fetus [5].

In our patient, the osseous component involved was the acetabulum, which was underdeveloped and defective. This can primarily be attributed to a defect in the proximal femoral epiphyseal plate as it plays an important role in the development of the hip joint. Gage et al. studied the effects of trochanteric epiphyseodesis on the growth of the proximal end of the femur following necrosis of the capital femoral epiphysis and concluded that insult to the epiphysis in infancy results in a mean growth loss in the proximal end of the femur of 21.5 millimeters with trochanteric epiphyseodesis [6].

Furthermore, as the femur is displaced superiorly in this case, the shear forces of muscles (traction epiphysis) generate pulling forces in angles different from normal. This changes the shape and growth of proximal femur and acetabulum, leading to the hip subluxation/dislocation. Furthermore, a pseudo acetabulum has most likely formed in order to stabilize the displaced femur and enable articulation. This pseudo acetabulum is made up of scar tissue and is further stabilized by muscles acting on the joint, the shadow of which is visible in Figure 1 outlined by the femoral head. As Odak et al. discuss in their paper, the presence of a well-defined, concentric, radio-opaque shadow around the dislocated femoral head is suggestive of a pseudo acetabulum [7]. Although this pseudo acetabulum in their case was formed following a total hip arthroplasty in an elderly patient with a history of recurrent dislocations, the characteristics of the radiological findings suggest a similar prognosis in our patient. Moreover, inappropriate posture and predominant weight bearing on the right side could also cause greater compression of the femoral shaft on the ipsilateral side, thinning of the compact bone, and widening of the medullary cavity. Ultimately these changes, combined with the plates that were removed from the right side, resulted in a widened and inappropriately angled femoral shaft that is weaker than normal as seen in Figure 1.

Lastly, as our patient is a 53-year-old post-menopausal woman, hormonal changes may have resulted in osteomalacia in the femoral shaft leading to the recent onset of pain in the hip region. According to the study by Ji et al., the mean age of natural menopause is 51 years and results in decreased production of estrogen, which impairs the normal bone turnover cycle [8].

As a result of the aforementioned late presentation of DDH in our patient, the treatment is very complicated with debatable prognosis. Ideally, early diagnosis of DDH (at or before six months of age) is extremely important for therapeutic success as Pavlik bracing would be a viable option. Pavlik bracing is a soft dynamic harness that could be used to treat DDH in infants up to six months old. It maintains hip in flexion and abduction [9]. Due to the age and high degree of dysplasia (Crowe type IV) in our patient, the only viable surgical intervention would be total hip replacement [10]. This would involve subtrochanteric shortening osteotomy: excision of femur 4 cm below the head bilaterally and attachment of head to the shaft. Also, the acetabular socket will have to be widened to enable articulation. Finally, a total hip replacement will be performed bilaterally with an appropriately sized socket (~38 mm) and femoral head (~22 mm).

## Conclusions

DDH in adults is extremely uncommon. Early diagnosis of DDH is extremely important for therapeutic success. The treatment dilemma with late presentation is very complicated with debatable prognosis. As our patient was able to ambulate and is managing her activities of daily living adequately, surgical intervention may potentially lead to a reduced quality of life. As the risks of surgery may outweigh the benefits at this stage, she can be conservatively managed without surgery or any additional treatment other than continuing physical therapy and over the counter analgesics with follow up as needed if symptoms worsen.
